# Eating Behaviours and Dietary Intake in Children and Adolescents: A Systematic Review

**DOI:** 10.1007/s13668-024-00544-w

**Published:** 2024-05-26

**Authors:** Ivie Maneschy, Andrea Jimeno-Martínez, María L. Miguel-Berges, Azahara I. Rupérez, Ana Daniela Ortega-Ramiréz, Guiomar Masip, Luis A. Moreno

**Affiliations:** 1 Growth, Exercise, Nutrition and Development (GENUD) Research Group, University of Zaragoza, C/Pedro Cerbuna 12, 50009 Zaragoza, Spain; 2 Instituto de Investigación Sanitaria de Aragón (IIS Aragón), Zaragoza, Spain; 3 Centro de Investigación Biomédica en Red de Fisiopatología de la Obesidad y Nutrición (CIBERObn), Instituto de Salud Carlos III, Madrid, Spain; 4 Instituto Agroalimentario de Aragón (IA2), 50009 Zaragoza, Spain; 5 Faculty of Medicine, University of Colima. Av. Universidad 33 Colonia Las Víboras, CP 28010 Colima, México

**Keywords:** Eating behaviour, Childhood, Adolescents, Dietary intake

## Abstract

**Purpose of Review:**

This systematic review aimed to examine existing evidence related to associations between eating behaviours and dietary intake in children and adolescents, with a focus on the Children Eating Behaviour Questionnaire (CEBQ) and the Dutch Eating Behaviour Questionnaire (DEBQ) as assessment tools.

**Recent Finding:**

We conducted a systematic review following PRISMA guidelines. We included observational and interventional studies published in English, Spanish, or Portuguese, that evaluated the association between eating behaviours and food and beverage intake. Thirteen studies from nine countries met the inclusion criteria, with sample sizes ranging from 62 to 4,914 individuals aged 2 to 16 years-old. Ten studies used the CEBQ, and three used the DEBQ. Our retrieved studies showed that children and adolescents engaging in food approach behaviours tend to consume foods rich in sugar and fats. However, we observed a higher consumption of fruits and vegetables. On the other hand, children and adolescents with lower engagement to food avoidant behaviours, generally exhibited a lower overall food consumption, except for snacks, which they consumed at a higher rate.

**Summary:**

This systematic review suggests that eating behaviours play an important role in shaping dietary intake. Nevertheless, due to the heterogeneity related to eating behaviours and diet intake, it highlights the need for further research to understand these complex relationships to develop effective interventions for promoting healthy eating habits in children and adolescents.

**Supplementary Information:**

The online version contains supplementary material available at 10.1007/s13668-024-00544-w.

## Introduction

Eating behaviour encompasses the way in which individuals eat, involving not only food choices but also the physiological processes associated with food intake, such as mastication and deglution [1, 2]. Eating behaviours are influenced by environmental, social and biological factors [3, 4]. Considering that eating behaviours influence food choices, it is essential to prioritize the study of eating behaviours during childhood and adolescence, crucial periods when they are still in developmental stages [5–7].

To assess eating behaviours in children, several questionnaires are available. However, the most widely used are the Children Eating Behaviour Questionnaire (CEBQ) and the Dutch Eating Behaviour Questionnaire (DEBQ). The CEBQ is a validated questionnaire that has been. translated into dozens of languages [8, 9], and comprises 35 questions evaluating eight different subscales. It aims to analyze food approach and food avoidance in children [3, 8]. The DEBQ, was originally designed to measure three eating behaviour subscales (emotional eating, external eating and restrained eating) in adults. Later, it was adapted for children aged seven to twelve years old, becoming the DEBQ-Children (DEBQ-C). This questionnaire comprises 20-items addressing emotional, restrained and external eating behaviours [10]. It is a validated instrument that has been translated into multiple languages [11–13].

According to the World Health Organization, the prevalence of overweight and obesity in children and adolescents has increased globally, representing a significant public health concern, with rates reaching a prevalence of 18%, encompassing both overweight and obesity and affecting both sex similarly [14]. In this context, food intake and dietary patterns have been suggested to have a significant impact on the development of childhood obesity [6, 15–17].

While it is widely recognized that eating behaviours may influence dietary intake, there are still gaps in our understanding of the specific associations between eating behaviours subscales and food and beverage intake in children and adolescents [18–21]. Although several studies examined these associations individually, systematic reviews addressing these relationships are lacking. To our knowledge, this is the first systematic review that specifically assess the associations between eating behaviours, as measured by the CEBQ [8] and DEBQ [10], and the consumption of foods and beverages during childhood and adolescence. Therefore, this systematic review aims to elucidate the relationship between eating behaviours and food and beverage intake in this population.

## Methods

We conducted a systematic review following the Preferred Reporting Item for Systematic Review and Meta-analysis (PRISMA) guidelines [22]. We registered the protocol in the International Prospective Register of Systematic Reviews (PROSPERO), registration number CRD42020153990.

### Search Strategy and Review Process

We conducted a comprehensive electronic systematic search using six electronic databases: MEDLINE via PubMed, Cochrane Library, EMBASE, Web of Science, Scientific Electronic Library Online (SciELO) and Scopus. The search was restricted to articles published until March 2020, and updated in November 2022 to include articles published up to that date. We used the combinations of relevant keywords and MESH terms, based on descriptors relative to the Population, Intervention, Control, Outcomes (PICO) principle [23] using the following search terms: ("eating behavior" OR “food behavior” OR “eating behaviors” OR “food behaviors” OR “eating behaviour” OR “food behaviour” OR “feeding patterns” OR “food habits”) AND ("energy intake" OR “food intake” OR “ingestion” OR “nutrient intake” OR “dietary intake”) AND (child* OR preschool* OR adolescent* OR teen* OR “young people” OR “youth”). We did not impose any restrictions based on publication date, except the ones mentioned above.

**Fig. 1:**
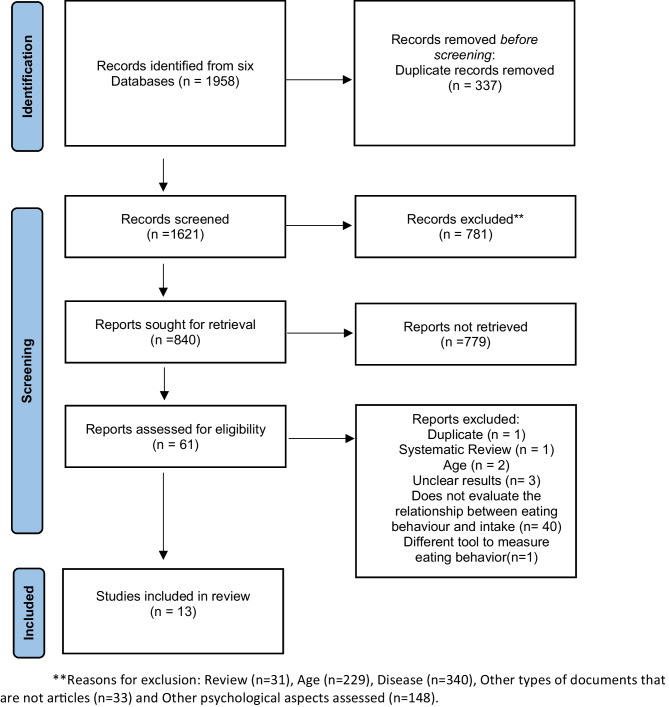
PRISMA 2020 flow diagram of the literature search and article selection

Figure 1 describes a flow diagram with the number of articles included and excluded during the systematic review process following the PRISMA guidelines.

### Inclusion and Exclusion Criteria of the Retrieved Studies

The search strategy was designed to capture all observational and interventional studies that evaluated associations between eating behaviours and food and beverage intake. Thus, in the present report articles were considered eligible if they met these inclusion criteria: a) peer-reviewed observational studies (i.e., cross-sectional studies, case-control studies, longitudinal studies) and intervention studies (i.e., randomized clinical), b) published in English, Spanish or Portuguese, c) conducted in children and/or adolescents aged 1 to 18 years old [24], d) reported food and beverage intake, and e) assessed associations between eating behaviours and foods and beverage intake.

Exclusion criteria comprised: 1) studies with participants with any disease, except obesity, 2) studies involving age groups other than individuals between 1 and 18 years, and 3) studies lacking a separate analysis by age group.

Studies were eligible for inclusion if they reported at least one characteristic measured by either CEBQ or DEBQ. The CEBQ comprises eight subscales: Enjoyment of Food, Food Responsiveness, Emotional Overeating, Desire to Drink, Food Fussiness, Emotional Undereating, Slowness in Eating and Satiety Responsiveness. The DEBQ assesses three subscales: Restrained, Emotional and External Eating Behaviour. Scale scores indicating a greater frequency of the being assessed. More details on questionnaires specifics and evaluation methods can be found in the original publications [8, 10]. While not an inclusion criterion, the food consumption assessment methods found in this systematic review predominantly included food frequency questionnaires (FFQ) and food records or diary.

### Data Extraction and Quality Assessment of the Retrieved Studies

The search and selection of studies was carried out in a blind manner by two authors (IM and AJ). Discrepancies were analysed and discussed with a third researcher (AR), to obtain consensus.

The following data were extracted from the retrieved studies: identification data including citation of the author(s), year of publication, and country; study design; language; age range; sex; method used to obtain food intake information; eating behaviour tools and subscales; and main results. Two authors (IM and AJ) assessed the risk of bias and the methodological quality of the articles using the National Heart Lung and Brain Institute (NHLBI) Study Quality Assessment tool [25]. This tool consists of 14 questions based on study design focusing on sampling methods, sample characteristics, participation rate, and analysis methods. Authors categorized each study as ‘Good’, ‘Fair’ or ‘Poor’ based on their responses and reported study limitations according to the guidance accompanying in the NHLBI quality assessment tool. Quality scores (Table 1) and limitations were not used to exclude studies. Details of the evaluation of each included study can be found in the Supplementary section, Table S1.

**Table 1:** Summary of characteristics and results of retrieved studies (n = 13)

**Participants**					**Eating Behaviour assessment**		**Outcome: Diet Intake**	
**Author, date, country**	**Study design**	QA overall result*	**Age range**	**N, gender %**	**Tool**	**Subscales**	**Dietary assessment**	**Results**
**Albuquerque et al., 2018** **Portugal** [26]	Cohort study	Good	4–7 years	4358, 49,3% F	CEBQ	Disinhibition: (FR, EOE, EUE, DD) Restraint: (SR, SE, FF, EF)	FFQ, 3-day food record Dietary patterns: Snaking / Energy dense food/ Healthier	Higher scores in Appetite Restraint and Appetite Disinhibition dimensions at age 7 were found in children who belonged to 'Snacking' and 'Energy dense food' patterns, respectively, at age 4, compared to children with the 'Healthier' pattern. Maternal BMI before pregnancy modified the 'Snacking' pattern; it was stronger in children of underweight/normal weight mothers for Appetite Restraint and present only among overweight/obese mothers for Appetite Disinhibition.
**Blissett et al., 2016** **England** [27]	Intervention/ RCT study	Fair	2–6 years	99 47,4% F	CEBQ	EF, FR, FF, SR	Fruit consumption Novel Fruit (NF): Dried date, tinned lychee and fresh fig	There was a main effect of FF on the percentage of NF consumed by the children (P = 0.008). Children with higher scores on FF consumed less NF.
**Blissett et al., 2019** **England** [18]	Intervention/ RCT study	Fair	3–5 years	62, 46,7% F	CEBQ	EF, FR, EOE, FF, EUE, SR	Snack Food consumption	Children who ranked higher on the EUE consumed less snacks/crisps and cookies. Children with higher FR consumed more breadsticks. Children with higher EF consumed more chocolate. Children who ranked higher on SR consumed more carrots but did not consume more or less of any other snack. Children who ranked higher on FF consumed more breadsticks.
**De Cock et al., 2016** **Belgium** [28]	Cross- sectional study	Fair	14- 16 years	1104, 49% F	DEBQ	Emotional and External	FFQ (Snacks and Sugar sweetened beverages)	Reward sensitivity was positively associated with consumption of unhealthy snacks. (P > 0.001). The relationship between Reward Sensitivity and consumption of unhealthy snacks was partially mediated by external (p < 0.05) and emotional (p < 0.01) eating.
**Carnell et al.,** **2016** [36]	Cross- sectional study	Fair	4–5 years	70, --	CEBQ	EF, FR, SE, SR	Food group Intake (Snacks, white bread Protein food, and Fruit and vegetables)	Children with higher SR showed a lower average consumption of all food categories (Snacks, white bread, protein food, fruit and vegetables). Children with a higher FR showed a higher consumption of white bread and fruits and vegetables. Children with higher EF showed a higher consumption of all categories (Snacks, white bread, protein food, fruit and vegetables). When analysing the percentage of consumption, children with higher EF ate more white bread and less snacks.
**Elfhag et al., 2007** **Sweden** [29]	Cross- sectional study	Fair	10- 13 years	1853, 51,05% F	DEBQ	Restrained, Emotional and External	Soft Drinks Consumption	Restrained eating showed opposite associations for the two types of soft drinks. Sugary soft drinks were related to a lower restrained eating score, while light soft drinks were related to a higher score. In girls, there was a small positive association between emotional eating and sugary soft drinks. External eating was more related to sugary soft drinks, except for boys, who had a small correlation between external eating and light soft drinks.
**Elfhag et al., 2008** **Sweden** [30]	Cross- sectional study	Fair	12 years	1441, 50,7% F	DEBQ	Restrained, Emotional and External	FFQ (Fruits, Vegetables, Sweets, Soft Drinks)	In boys, restrained eating was inversely associated with consumption of sweets. In girls, Emotional eating was positively associated with consumption of sweets and soft drinks. External eating was related with the consumption of sweets.
**Holley et al., 2018** **England** [37]	Cross- sectional study	Fair	2- 5 years	150, --	CEBQ	EF, FR, FF, SE	FFQ (vegetables consumption)	Children with a high FF score showed a lower weekly vegetable consumption (r = − 0.42, p < 0.001). High FR and EF scores were negatively associated with difficulty for eating vegetables. FF and SE were positively associated with difficulty for eating vegetables.
**Jalkanen et al., 2017** **Finland** [32]	Intervention/ RCT study	Fair	6- 8 years	406, 50,2% F	CEBQ	EF, FR, EOE, DD, FF, EUE, SE, SR	4- day food diary (food group Intake)	A higher score on the EF subscale was associated with higher vegetables consumption, higher cheese consumption, and higher meat consumption. A higher FR score was associated with higher fruits and berries consumption and higher meat consumption.
**Sandvik et al., 2019** **Sweden** [33]	Intervention/ RCT study	Fair	4- 6 years	130, 41,5% F	CEBQ	FF	FFQ (food group Intake)	FF was negatively associated with vegetables intake.
**Tharner et al., 2014** **Netherlands** [34]	Cohort study	Good	4 years	4914, 50% F	CEBQ	EF, FR, FF, SE, SR ("fussy" eating behaviour profile (picky eaters) characterized by high FF, SE and SR in combination with low EF and FR was computed)	FFQ (food group Intake)	Children identified as picky eaters at age four ate fewer whole grain products (mean difference = 0.28 SD, p < 0.01), less vegetables (mean difference = 0.20 SD, p < 0.05), less fish/seafood (mean difference = 0.16 SD, p < 0.05) and less meat (mean difference = 0.22 SD, p < 0.05) at 14 months of age than children later identified as not demanding eaters. At 14 months, fussy eaters consumed more salty snacks (mean difference = 0.10 SD, p < 0.05) and confectionery (mean difference = 0.19 SD, p < 0.05) than the non-picky eaters.
**Vilela et al., 2019** **Portugal** [19]	Cohort study	Good	4- 7 years	1359, 47,3% F	CEBQ	EF, FR, DD, SE, SR	3- day food diary	In the cross-sectional analysis at 7 years of age, and after multivariate adjustment, SR was positively associated, and EF negatively associated, with consumption of daily snacks. DD was positively associated with greater consumption of daily snacks at age 4. A higher consumption of daily snacks at age 7 was positively associated with SE (β = 0.056, 95% CI: 0.001; 0.112) only after adjusting for energy intake.
**Wild et al., 2018** **Denmark, Greece and the Netherlands** [35]	Intervention/ experimental study	Fair	2- 6 years	750, 47, 8% F	CEBQ	FF, EF, SR, FR	Vegetables consumption	FF and EF (P < 0.001) were positively associated with children's vegetables intake, but only in the Netherlands.

*BMI* body mass index, *CEBQ* Child Eating Behaviour Questionnaire, *CI* confidence interval, *DD* Desire to Drink, *DEBQ* Dutch Eating Behaviour Questionnaire, *EF* Enjoyment of Food, *EOE* Emotional Overeating, *EUE* Emotional Undereating, *FFQ* Food Frequency Questionnaire, *FF* Food Fussiness, *FR* Food Responsiveness, *hr* hours, *Kcal* Kilocalories, *Q.A* Quality Assessment, *NF* Novel Fruit, *RCT* Randomized controlled trial, *SE* Slowness in Eating, *SR* Satiety Responsiveness

*The risk of bias and the methodological quality of the articles were assessed using the National Heart Lung and Brain Institute (NHI) Study Quality Assessment tool (Study Quality Assessment Tools | NHLBI, NIH, s. f.)

### Heterogeneity Assessment

Due to the variation in study characteristics, particularly in study designs and tools for measuring food and beverage intake, conducting a meta-analysis was deemed unfeasible. Therefore, the results of this systematic review were analysed narratively.

## Results

A total of 1,958 articles were identified across the six databases. After removing duplicates 337 articles were excluded and 1,560 were excluded based on title and abstract screening (781 after title screening and 779 after abstract screening). From the remaining 61 articles selected for full-text reading, 13 met the inclusion criteria and were included in this systematic review.

Table 1 presents the characteristics of the retrieved studies and their main results. The retrieved studies were conducted in nine countries from two continents: Europe (12 out of 13, 92.3%) [18, 19, 26–35] and America 1/13 (7.7%) [36]. Regarding study design, eight were cross-sectional or cohort studies, while five were intervention studies (4 randomized clinical trials and 1 other intervention study with no standardized methodology). The sample sizes ranged from 62 to 4,914 individuals, with ages between two to sixteen years old.

Among the included studies, 10 used the CEBQ, while three used the DEBQ. Due to a large heterogeneity in the analysed food and beverage groups and assessment tools, results are presented as either positive or negative associations between eating behaviours and the consumption of various foods and beverages. The most frequently evaluated food and beverage groups included snacks, fruits, vegetables, sweets, and sugar-sweetened beverages (SSB).

### Studies Assessing Eating Behaviours Using the CEBQ

Regarding instruments used to assess food and beverage intake, six studies used FFQs, three studies used food diaries, and five studies evaluated the consumption of specific food and beverage groups without using a standardized instrument or specifying the instrument in the article. Two studies evaluated all subscales of the CEBQ, while the remaining studies evaluated groups of selected subscales. Specifically, seven studies evaluated Food Responsiveness, Enjoyment of Food was evaluated in seven studies, Satiety Responsiveness in six studies, Food Fussiness in six studies, Slowness in Eating in four studies, Emotional Overeating in one study, Desire to Drink in one study, and Emotional Undereating in one study.

In the 10 studies that reported associations between CEBQ subscales and food and beverage intake, the food approach (Enjoyment of Food, Food Responsiveness, Emotional Overeating and Desire to Drink) and food avoidant (Slowness in Eating, Satiety Responsiveness, Food Fussiness, and Emotional Undereating) subscales consistently showed associations with food and beverage consumption in the expected direction.

These studies evaluated the consumption of various food groups including snacks, fruits and vegetables, breads, cheese and meats. The analyses revealed positive associations for Enjoyment of Food (in 6 out of 9 studies, 67%), Food Responsiveness (in 5 out of 9 studies, 56%), Satiety Responsiveness (in 4 out of 8 studies, 50%), Food Fussiness (in 3 out of 8 studies, 38%), Slowness in Eating (in 1 out of 6 studies, 17%) Desire to Drink (in 1 out of 3 studies, 33%), as well as negative associations for Enjoyment of Food (in 1 out of 9 studies, 11%), Food Responsiveness (in 1 out of 9 studies, 11%), Satiety Responsiveness (in 1 out of 8 studies, 13%), Emotional Undereating (in 1 out of 3 studies, 33%) Slowness in Eating (in 3 out of 6 studies, 50%), and Food Fussiness (in 3 out of 8 studies, 38%).

Nine studies evaluated the food approach subscales, with six (67%) showing significant positive associations between Enjoyment of Food and food intake, and five (56%) showing significant associations for Food Responsiveness and food intake. Specifically, significant positive associations were showed between Enjoyment of Food and the intake of fruits, vegetables, white bread, cheese, meat and chocolate. Similarly, significant positive associations were shown between Food Responsiveness and the intake of fruits and berries, vegetables, snacks, meat and white bread.

**Fig. 2:**
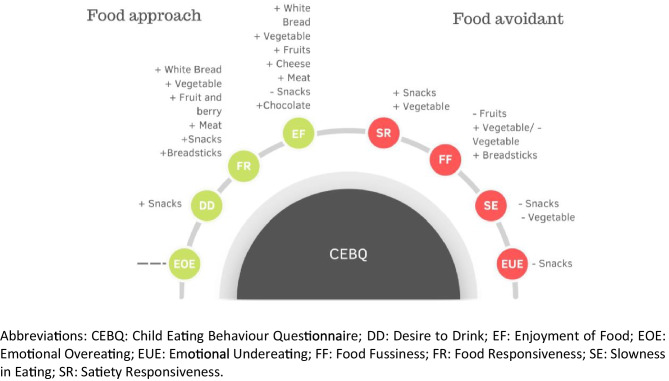
Overview of the results of the studies that used de CEBQ tool (n = 10) included in this systematic review

A total of 11 studies assessed at least one food avoidant subscale, of which three studies reported a negative association between Food Fussiness and vegetable intake. However, de Wild et al*.* [35], showed the opposite, a positive association. Results were inconclusive for Satiety Responsiveness and Slowness in Eating; higher scores in these subscales were related to lower food consumption but higher daily snacking. Only one study showed a negative association between Emotional Undereating and the consumption of some snacks. Table 1 presents descriptive summaries of these relationships, in addition to a visual summary in Fig. 2.

### Studies Assessing Eating Behaviours Using the DEBQ

Three studies used the DEBQ as a tool to measure eating behaviour. Among these studies, two utilized all three subscales: emotional, external and restrained eating, while one study did not include the restrained eating subscale.

Similar to other studies included in this systematic review, different tools were used to assess food consumption. Specifically, two studies used FFQs, and one study used a food diary. Different food groups were evaluated, including sweets, soft drinks and snacks.

**Fig. 3:**
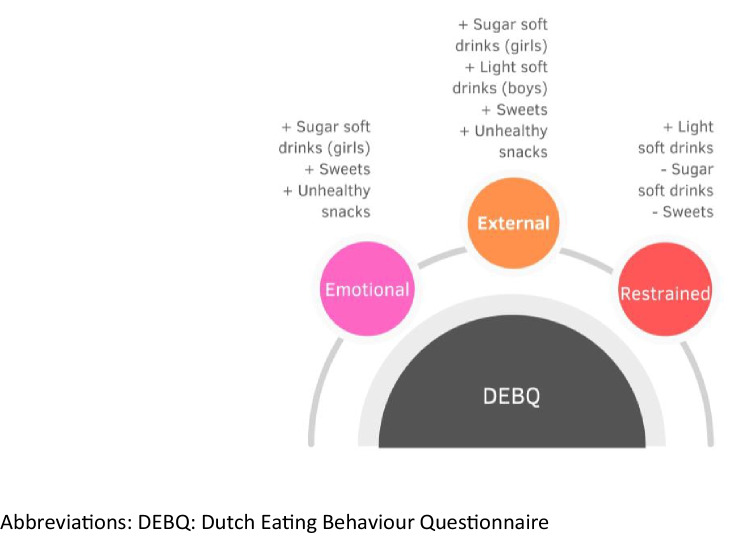
Overview of the results of the studies that used the DEBQ tool (n = 3) included in this systematic review

Positive associations were reported for emotional eating (3 out of 3, 100%) and external eating (3 out of 3, 100%), while negative associations were showed for restrained eating (2 out of 3, 67%). Particularly, positive associations were shown between emotional eating and the consumption of unhealthy snacks, sweets and soft drinks (only observed in girls). On the other hand, a negative association was observed between restrained eating and the consumption of sweets and soft drinks. For external eating, positive associations were shown with the consumption of sweets, unhealthy snacks, sugary soft drinks (observed only in girls) and light soft drinks (observed only in boys). Descriptive summaries of these relationships are presented in Table 1, in addition to a visual summary in Fig. 3.

## Discussion

The aim of the current systematic review was to analyse the existing literature on the relationship between eating behaviours and dietary habits during childhood and adolescence. Our findings provide information on how eating behaviours are associated with food and beverage intake in the study population. We focused on two widely-used and validated questionnaires, the CEBQ and the DEBQ, which have been extensively employed in various countries. Notably, this systematic review represents the first comprehensive analyses of the associations between eating behaviours and food and beverage intake in children and adolescents. This systematic review included a total of 13 studies that assessed eating behaviours using the CEQB and DEBQ, along with food and beverage intake in children and adolescents. Most of the retrieved studies used the CEBQ and its subscales. The findings consistently demonstrated a significant association between eating behaviours and food consumption.

When analysing food approach subscales, this systematic review identified significant associations between the Enjoyment of Food subscale and the consumption of fruits, vegetables, white bread, cheese, meat and chocolate. Similarly, Food Responsiveness was associated with the consumption of fruits and berries, vegetables, snacks, meat and white bread. These findings are consistent with other studies that have reported a positive association between Enjoyment of Food and Food Responsiveness with vegetable consumption [34, 35, 37]. Furthermore, two other studies [32, 36] found a positive association between Food Responsiveness and the intake of fruits and vegetables.

In the analysis of studies utilizing the CEBQ, we found that Satiety Responsiveness was associated to a lower consumption of snacks [19, 36], whereas Enjoyment of Food and Food Responsiveness were associated with a higher snack consumption [18, 19, 36]. Studies investigating Enjoyment of Food and Food Responsiveness have found that higher scores on these subscales were associated to increased overall food intake. Two studies found that individuals with higher Enjoyment of Food and Food Responsiveness scores tended to have a higher frequency of daily meals [19, 32], suggesting they have an increased energy intake. Additionally, parental modelling has been observed to influence children’s intake of new types of fruits, even among those children who may have lower scores in food approach subscales [27]. Indicating that food parenting behaviours play a role in influencing children’s dietary habits, including their willingness to try new foods.

In summary, high scores on engaging food approach subscales were consistently associated with increased consumption of fruits and vegetables. On the other hand, this systematic review also revealed an association between high scores on engaging food approach subscales and the consumption of energy-dense foods, such as sweets, sugar-sweetened beverages and snacks. While individuals exhibiting food approach behaviours tend to have a greater risk of developing obesity and overweight [3], it is important to recognize that these subscales are also associated with increased consumption of health foods such as fruits and vegetables. This underscores the complexity of the relationship between food approach eating behaviours and weight status. Further research is needed to understand the underlying mechanisms driving these differences in food choices and their implications for weight outcomes.

Significant associations were found between higher engagement in food avoidant behaviours and dietary intake. Food Fussiness was associated with lower consumption of vegetables [33–35, 37], as well as other food groups such as meat, fish and whole grains [34]. Moreover, one study also indicated that children exhibiting Food Fussiness consumed fewer new types of fruits, including dried dates, tinned lychee and fresh figs [27]. Additionally, children who exhibited higher levels engagement in Slowness in Eating and Satiety Responsiveness showed reduced intake of vegetables and overall food consumption. This pattern was associated with increased daily snacking [19]. Further, engaging in Slowness in Eating, was associated with a reduced vegetable consumption [34, 37]. Children and adolescents engaging in Food Fussiness behaviour showed an increased consumption of snacks [18, 36]. Another study also showed a similar association, indicating that engagement in Desire to Drink and Slowness in Eating behaviours were associated to high snack consumption [19]. Conversely, children and adolescents engaging in Emotional Undereating exhibited decreased consumption of snacks [18]. Interestingly, in the same study, children with the highest engagement in Satiety consumed more snacks, particularly carrots [18]. Highlighting the need for further studies analysing the quality of the snacks and how their consumption may influence the association with various Food Avoidant behaviours.

Other research has explored how various combinations of subscales from the CEBQ can characterize dietary habits in children. For example, one study grouped five subscales to create a fussy eating pattern, combining high scores in Food Fussiness, Slowness in Eating and Satiety Responsiveness with low scores in Enjoyment of Food and Food Responsiveness. The findings revealed that children identified as picky eaters at four years of age consumed fewer whole grains, vegetables, fish and /or seafood, and meat during infancy, when they were 14 months old, compared to non-picky eaters at the same age. Additionally, fussy eaters at 14 months old had higher intake of snacks compared to non-fussy eaters [34]. In another study from Albuquerque et al., categorized CEBQ subscales into two groups: restraint and disinhibition. The restraint group comprised Satiety Responsiveness, Food Fussiness, Slowness in Eating, Enjoyment of Food, while the disinhibition group included Food Responsiveness, Emotional Overeating, Emotional Undereating and Desire to Drink. In this study, they found that both groups showed higher consumption of snacks and energy-dense foods [26]. The elevated intake of these ultra-processed foods is associated with the risk of developing metabolic diseases in the future [38, 39], underscoring the involvement of both food approach and food avoidant in this less healthy dietary intake pattern.

In this systematic review, we observed different associations between various eating behaviours subscales and dietary intake. For instance, high scores on food approach behaviours, such as Enjoyment of Food and Food Responsiveness, were associated with increased consumption of fruits and vegetables, but also with energy-dense foods. Conversely, engagement in food avoidant behaviours was associated with lower intake of vegetables, as well as increased consumption of snacks. These discrepancies may be attributed to potential overlap between some scales, such as Food Responsiveness and Enjoyment of Food or Satiety Responsiveness and Slowness in Eating. Those subscales showing stronger associations are likely to exhibit higher frequencies of observed outcomes when compared to other subscales [9, 40].

Retrieved studies using the DEBQ tool and its external and emotional eating subscales has demonstrated a direct association with increased consumption of snacks [28], sweets and sugar-sweetened beverages [29, 30] in children and adolescents and, with sugar-sweetened beverage consumption associated specifically to girls. Conversely, on the restrained eating scale, a lower score was associated with greater consumption of sweets [28].

In this systematic review, several limitations must be acknowledged. Firstly, the absence of a standardized tool for assessing dietary intake across studies is challenging. While some used The FFQs to capture long-term dietary consumption patterns [41], a recent meta-analyses highlighted the FFQs limitations in accurately estimating dietary intake among children and adolescents [42]. Nevertheless, we included studies using FFQs, due to their capacity of assessing large periods of time, usually 1 year. Secondly, the limited number of intervention studies presented challenges for result comparison, given the diverse nature of intervention. Moreover, certain studies did not establish direct associations between eating behaviours and food and beverage intake, as they incorporated additional behavioural and parenting variables. Additionally, modifications to the original formats of the CEBQ or DEBQ in some studies, where subscales were combined to create new pattern of behaviour patterns, hindered result standardization. Despite these limitations, our systematic reviews’ strengths lie in the standardization of the sample population by age, exclusively comprising children and adolescents. Furthermore, this systematic review emphasizes the importance of studying eating behaviours within food and beverage consumption, considering social, environmental and psychological factors that may influence food choices.

## Conclusions

This systematic review provides evidence supporting the relationship between eating behaviours and food and beverage intake in children and adolescents. Higher engagement in food approach behaviours and lower engagement in food avoidant behaviours were associated with increased consumption of foods such as white bread, cheese, meat, and chocolate and snacks, which may potentially increase the risk of developing obesity during childhood and adolescence. However, it is noteworthy that food approach behaviours also demonstrated a high consumption of healthy foods, including vegetables and fruits. Understanding eating behaviours may help in preventing diseases related to food intake and body composition [43], highlighting specific targets for intervention. More longitudinal studies are needed to assess causality and explore bidirectional relationships, using validated food and beverage intake methods adapted to each country’s context. Nevertheless, this systematic review represents the most comprehensive analysis to date of research on the relationship between eating behaviours and food and beverage intake in children and adolescents. Recognizing that eating behaviours can have both positive and negative influences in food intake in children and adolescents, is crucial for fostering healthy eating habits in both parents and their offspring. These findings contribute to the existing evidence base and emphasize the importance of eating behaviours in food and beverage selection and the prevention of non-communicable diseases, such as obesity.

### Supplementary Information

Below is the link to the electronic supplementary material.

Supplementary file1 (DOCX 23 KB)

## Data Availability

No datasets were generated or analysed during the current study.

## List of Abbreviations

CEBQ: Children Eating Behaviour Questionnaire
DEBQ: Dutch Eating Behaviour Questionnaire
FFQ: Food Frequency Questionnaire
