# Supplementary material for: Eating Behaviours and Dietary Intake in Children and Adolescents: A Systematic Review
Source: Curr Nutr Rep. 2024 May 26;13(3):363–76. doi: 10.1007/s13668-024-00544-w (PMC11327180; doi:10.1007/s13668-024-00544-w)
Supplement: Supplementary file 1 — Supplementary file1 (DOCX 23 KB) [file 13668_2024_544_MOESM1_ESM.docx]

**Supplementary table 1.** Quality assessment for Observational Cohort and Cross-Sectional Studies.

| **Studies** | **Quality assessment criteria^a^** | | | | | | | | | | | | | | **Quality score (%)** |
| --- | --- | --- | --- | --- | --- | --- | --- | --- | --- | --- | --- | --- | --- | --- | --- |
|  | **1** | **2** | **3** | **4** | **5** | **6** | **7** | **8** | **9** | **10** | **11** | **12** | **13** | **14** |  |
| Albuquerque et al. 2018 | Y | Y | Y | NR | NA | NR | Y | Y | Y | NR | Y | NR | Y | Y | **9** |
| De Cock et al. 2016 | Y | Y | Y | NR | Y | Y | NA | NA | Y | NA | Y | NR | NA | Y | **8** |
| Carnel et al. 2016 | Y | Y | N | Y | NR | Y | NA | NA | Y | NA | Y | NR | NA | N | **6** |
| Elfhag et al. 2008 | Y | Y | N | Y | NR | Y | NA | NA | Y | NA | Y | NR | NA | NR | **6** |
| Elfhag et al. 2007 | Y | Y | Y | Y | NR | NR | NA | NA | Y | NA | Y | NA | NA | Y | **7** |
| Holley et al. 2018 | Y | Y | NR | Y | Y | Y | NA | NA | Y | NA | Y | NA | NA | NR | **7** |
| Jalkenen et al. 2017 | Y | Y | Y | Y | NR | NR | NA | NA | Y | NA | Y | NA | NA | NR | **6** |
| Tharner et al.2014 | Y | Y | Y | Y | Y | Y | Y | NR | Y | N | Y | NR | N | NR | **9** |
| Vilela et al. 2019 | Y | Y | Y | Y | NR | Y | Y | NR | Y | Y | Y | NR | N | Y | **10** |

CD, cannot determine; NA, not applicable; NR, not reported ^a^ (1) Was the research question or objective in this paper clearly stated? (2) Was the study population clearly specified and defined? (3) Was the participation rate of eligible persons at least 50%? (4) Were all the subjects selected or recruited from the same or similar populations? Were inclusion and exclusion criteria for being in the study prespecified and applied uniformly to all participants? (5) Was a sample size justification or power description provided? (6) For the analyses in this paper, were the exposure(s) of interest measured prior to the outcome(s) being measured? (7) Was the timeframe sufficient so that one could reasonably expect to see an association between exposure and outcome if it existed? (8) For exposures that can vary in amount or level, did the study examine different levels of the exposure as related to the outcome (e.g. categories of exposure or exposure measured as continuous variable)? (9) Were the exposure measures (independent variables) clearly defined, valid, reliable, and implemented consistently across all study participants? (10) Was the exposure(s) assessed more than once over time? (11) Were the outcome measures (dependent variables) clearly defined, valid, reliable, and implemented consistently across all study participants? (12) Were the outcome assessors blinded to the exposure status of participants? (13) Was loss to follow-up after baseline 20% or less? (14) Were key potential confounding variables measured and adjusted statistically for their impact on the relationship between exposure(s) and outcome(s)?

**Supplementary table 2.** **Quality Assessment Tool for Before-After (Pre-Post) Studies With No Control Group**.

| **Studies** | **Quality assessment criteria^a^** | | | | | | | | | | | | | | | | | **Quality score (%)** |
| --- | --- | --- | --- | --- | --- | --- | --- | --- | --- | --- | --- | --- | --- | --- | --- | --- | --- | --- |
|  | **1** | **2** | | **3** | **4** | **5** | **6** | **7** | **8** | **9** | | | **10** | **11** | **12** |  |  |  |
| Blissett et al. 2016 | Y | Y | NR | | N | NR | Y | Y | N | | Y | Y | | NR | NR |  |  | **6** |
| Blissett et al. 2019 | Y | NR | NR | | Y | NR | Y | Y | N | | Y | Y | | NR | NR |  |  | **6** |
| Sandvik et al. 2019 | Y | Y | NR | | Y | NR | Y | Y | N | | NR | Y | | Y | NR |  |  | **7** |
| Wild et al. 2018 | Y | NR | NR | | Y | Y | Y | Y | N | | N | Y | | Y | NR |  |  | **7** |

^a^ Criteria Yes No; CD, cannot determine; NA, not applicable; NR, not reported * (1) Was the study question or objective clearly stated? (2) Were eligibility/selection criteria for the study population prespecified and clearly described? (3) Were the participants in the study representative of those who would be eligible for the test/service/intervention in the general or clinical population of interest? (4) Were all eligible participants that met the prespecified entry criteria enrolled? (5) Was the sample size sufficiently large to provide confidence in the findings? (6) Was the test/service/intervention clearly described and delivered consistently across the study population? (7) Were the outcome measures prespecified, clearly defined, valid, reliable, and assessed consistently across all study participants? (8) Were the people assessing the outcomes blinded to the participants' exposures/interventions? (9) Was the loss to follow-up after baseline 20% or less? Were those lost to follow-up accounted for in the analysis? (10) Did the statistical methods examine changes in outcome measures from before to after the intervention? Were statistical tests done that provided p values for the pre-to-post changes? (11) Were outcome measures of interest taken multiple times before the intervention and multiple times after the intervention (i.e., did they use an interrupted time-series design)? (12) If the intervention was conducted at a group level (e.g., a whole hospital, a community, etc.) did the statistical analysis take into account the use of individual-level data to determine effects at the group level?
